# Improvements in Blood Pressure Among Undiagnosed Hypertensive Participants in a Community-Based Lifestyle Intervention, Mississippi, 2010

**DOI:** 10.5888/pcd11.130269

**Published:** 2014-04-03

**Authors:** Jamie Zoellner, Jessica L. Thomson, Alicia S. Landry, Charkarra Anderson-Lewis, Carol Connell, Elaine Fontenot Molaison, Kathleen Yadrick

**Affiliations:** Author Affiliations: Jessica L. Thomson, US Department of Agriculture, Agricultural Research Service, Stoneville, Mississippi; Alicia S. Landry, Carol Connell, Elaine Molaison, Kathleen Yadrick, The University of Southern Mississippi, Hattiesburg, Mississippi; Charkarra Anderson-Lewis, University of Florida, Gainesville, Florida.

## Abstract

**Introduction:**

Effective strategies are needed to reach and treat people who lack awareness of or have uncontrolled hypertension. We used data from a community-based participatory research initiative, Hub City Steps, to quantify the prevalence of undiagnosed hypertension and determine the relationship between hypertension status at baseline and postintervention improvements in blood pressure and health-related quality of life.

**Methods:**

Hub City Steps was a 6-month preintervention–postintervention lifestyle intervention targeting hypertension risk factors. Outcome measures were collected at baseline, 3 months, and 6 months. Generalized linear mixed models were used to test for effects by time and hypertension status.

**Results:**

Of the enrolled sample (N = 269), most were overweight or obese (91%), African American (94%), and women (85%). When considering hypertension status, 42% had self-reported diagnosis of hypertension (self-reported subgroup; 84% with antihypertensive medication use); 36% had no self-reported medical history of hypertension, but when blood pressure was measured they had a clinical diagnosis of prehypertension or hypertension (undiagnosed subgroup); and 22% had no self-reported or clinical hypertension diagnosis (no hypertension subgroup). From baseline to 6 months, systolic blood pressure significantly improved for participants with self-reported hypertension [8.2 (SD, 18.2) mm Hg] and undiagnosed hypertension [12.3 (SD, 16.3) mm Hg], with undiagnosed participants experiencing the greatest improvements (*P* < .001). Effects remained significant after controlling for covariates. Health-related quality of life significantly improved for all 3 hypertension subgroups, with no apparent subgroup differences.

**Conclusion:**

This study reveals advantages of a culturally appropriate community-based participatory research initiative to reach those with undetected hypertension and effectively improve blood pressure status and health-related quality of life.

## Introduction

The overall prevalence of hypertension in the United States is estimated at 34%, with a disproportionate prevalence among African Americans (44%) (1). Hypertension is a leading risk factor for cardiovascular and cerebrovascular events and a leading cause of morbidity and mortality (1,2). Furthermore, annual health-care expenditures for hypertension are approximately $131 billion (3).

To complicate issues related to the high prevalence, racial disparities, cultural disparities, and higher health care costs, hypertension has also been termed a silent epidemic. Because hypertension can be asymptomatic, people often do not seek medical care, leaving it undetected and untreated. Likewise, even among those who are aware and treated, rates for uncontrolled hypertension are high. For example, the Centers for Disease Control and Prevention (CDC) examined awareness and pharmacologic treatment of uncontrolled hypertension (4) in the United States. Using National Health and Nutrition Examination Survey (NHANES) data sets and weighted population counts for 2003 through 2010, an estimated 66.9 million adults have hypertension, of which 35.8 million (54%) have uncontrolled hypertension. Among those with uncontrolled hypertension, an estimated 39% are not aware of their hypertension status, 16% are aware but untreated, and 45% are aware and treated.

Hub City Steps was a lifestyle intervention targeting hypertension risk factors in Hattiesburg, Mississippi. Hattiesburg includes about 47,000 residents, of which approximately 53% are African Americans and 42% are whites (5). Data from NHANES and Behavioral Risk Factor Surveillance System indicate that the prevalence of hypertension is 44% for women and 40% for men in Forrest County (6). Likewise, hypertension unawareness is approximately 18% among both women and men (6).

All phases of Hub City Steps were guided by community-based participatory research (CBPR) principles (7,8). CBPR aims to build equitable community–academic partnerships and promote community participation in all aspects of the research process (7,8). CBPR is recognized as a key strategy for translating research into practice that can help in reducing health disparities (9). Overarching research goals of this CBPR initiative were to 1) develop and assess community capacity to promote physical activity and healthy food choices (10), 2) test treatment effects of a 6-month CBPR lifestyle intervention on systolic blood pressure (SBP) among community participants (11,12), and 3) test the dose effects of 4 versus 10 follow-up motivational interviewing contacts on SBP over a 12-month maintenance phase. Given the emphasis on overall health within the Hub City Steps intervention, another objective of this CBPR initiative was to understand health-related quality of life (HRQOL) (13).

We sought to quantify the prevalence of undiagnosed hypertension and determine the relationship between hypertension status at baseline and postintervention improvements in blood pressure using data at 3 and 6 months. We focus on 3 subgroups, those with 1) self-reported diagnosis of hypertension, with or without antihypertensive medication use (self-reported subgroup); 2) no self-reported medical history of hypertension, yet measured clinical diagnosis of pre-hypertension or hypertension (ie, SBP greater than 120 or diastolic blood pressure [DBP] greater than 80 mm Hg) (undiagnosed subgroup); and 3) no self-reported or clinical diagnosis of hypertension (no hypertension subgroup). A secondary aim was to examine changes in HRQOL.

## Methods

This research was approved by the University of Southern Mississippi’s Institutional Review Board. Before study enrollment, written informed consent was obtained from participants. Guided by CBPR principles, a community advisory board composed of 21 local stakeholders, 8 academic members, and 3 community intervention staff was organized to advise on all aspects of the study design and implementation. Details on the composition and role of the community advisory board have been previously published (10,11). A series of 4 community conversations were also held with 25 community members. The goal of these conversations was to elicit input on several aspects of intervention planning, including recruitment of walking coaches and participants, retention of participants, intervention design, scheduling and format of education sessions, and data collection procedures. Summaries were provided to the research staff, and recommendations were incorporated into the research protocols. 

### Study design

The first phase of Hub City Steps was a 6-month preintervention–postintervention experimental intervention targeting hypertension risk factors conducted during January through August 2010. Theory and methods are described elsewhere (11,12). In brief, Hub City Steps integrated concepts from several theoretical frameworks including social support (14), self-determination theory (15), and the transtheoretical model of change (16). The lifestyle intervention consisted of 4 primary components. The first component was 3 sessions of one-on-one motivational enhancement, a type of motivational interviewing that uses a feedback approach (17). These 20-minute sessions were provided by trained intervention staff at baseline, 3-month, and 6-month data collection points. The second component consisted of social support provided by walking group volunteer leaders who were community members designated as “coaches.” These coaches were trained to encourage walking, goal setting, and submission of pedometer diaries within walking groups. Coaches also served as liaisons between participants and research staff. The third component consisted of self-monitoring with a pedometer diary. Participants were asked to wear the provided pedometer (Yamax model SW-701, Yamax Corporation, Tokyo, Japan) on the waist during waking hours and record their daily steps on weekly pedometer diary (postage-paid postcards) or by logging in to the intervention’s website. The fourth component consisted of five 90-minute monthly education sessions that included principles of the Dietary Approaches to Stop Hypertension (DASH) (18) and incorporated group physical activity and sharing of successes and challenges.

### Recruitment and eligibility

The research goal of this lifestyle intervention and primary outcome of interest was blood pressure reduction. Yet in working through the CBPR process in coordination with the mayor’s Get Healthy Hattiesburg initiative, it was apparent that restricting intervention participation to people with a medical hypertension diagnosis would exclude community members who also could benefit from the lifestyle intervention. For this reason, eligibility and recruitment efforts were aimed at reaching the broader community by emphasizing health and social opportunities of participating in Hub City Steps. Benefits included forming community walking groups and using local health promotion resources, such as walking tracks and trails in parks and other locations. Hub City Steps was widely publicized in Hattiesburg by using flyers, local media coverage (radio, television, newspaper), and word of mouth. Community awareness was further aided through strategic efforts promoting visibility at community and civic events (eg, the Hattiesburg Council of Neighborhoods quarterly meeting and the city-sponsored annual Night Out Against Crime community kick-off). Coaches were directed to recruit 10 to 12 participants for their team, and this emerged as the primary method by which participants were enrolled. Coaches were encouraged to communicate both the social and broad health benefits of participating in Hub City Steps. Although recruitment efforts were primarily directed toward African American residents, race or ethnicity was not an exclusion criterion. Eligibility criteria included 18 years of age or older, English speaking, noninstitutionalized, and resident of the Hattiesburg area, without regard to hypertension status or antihypertensive medication use. However, for safety reasons, screened individuals with blood pressure of 180/110 mm Hg or higher were directed to obtain immediate medical attention and were disqualified from participating.

### Outcome measures

Outcome measures were collected at the baseline, 3 months, and 6 months. The primary blood pressure outcomes were measured using standardized assessment procedures with an Omron HEM-907XL automatic inflation sphygmomanometer (Omron Group, Lake Forest, Illinois). The Omron was programmed to take 2 blood pressure measurements at a 1-minute interval and display the average reading. The measure was repeated a second time, 2 minutes after the first reading, and if the 2 average readings were within 10 mm Hg, the lowest reading was recorded; if not, a third measure was taken. Participants were instructed to have no caffeine, tobacco, or exercise for 1 hour before blood pressure assessment. Questionnaires were used to assess demographics, medical history, medication use, fasting, and smoking. A single item for perceived general health from the CDC’s Healthy Days measure was used to measure HRQOL (19). Other anthropometric, biological, and behavioral outcomes were collected and are reported elsewhere (11). Participants were compensated $15, $20, and $25, respectively, for their time involved in data assessments at the 3 successive time points.

### Data analyses

All statistical analyses were performed using SAS software, version 9.3 (SAS Institute Inc, Cary, North Carolina). The significance level of the tests was set at .05. Descriptive statistics were used to summarize demographic characteristics by hypertension status. Chi-squared tests of association (categorical variables) and analysis of variance (continuous variables) were used to compare baseline demographic characteristics among hypertension status subgroups.

Generalized linear mixed models, using maximum likelihood estimation, were used to test for effects of time and hypertension status on blood pressure and HRQOL. Maximum likelihood estimation was used to handle missing data in the repeated measures (20). Because of recognized limitations of computational complexity and relative instability of multiple imputations (20), imputations of missing data were not performed. Time (3 months and 6 months) was modeled as a repeated measure by using a variance components covariance matrix structure. We first modeled blood pressure changes by using time and hypertension status only as predictor variables, initially including all participants with available data and then excluding those who reported a medication change. We subsequently included demographic and baseline variables (ie, age, marital status, educational attainment, income status, and baseline body mass index) to determine if hypertension status remained a significant predictor of blood pressure changes in the presence of covariates. An interaction term for age and hypertension status was also included because of the age differences apparent among the hypertension subgroups at baseline. Race, sex, and smoking status were not included in these models because of the low proportions of men, current smokers, and races other than African American. Interpretation of results based on such small subsets of participants is difficult and can be misleading. Least squares means were computed to estimate and compare blood pressure changes, with Tukey-Kramer adjusted *P* values used for multiple group comparisons. This study was powered (80% power; α of .05) to detect a moderate effect size of 0.4 (difference of 6 [SD, 15] mm Hg between groups) for SBP at 18 months while controlling for 6-month intervention treatment effects.

## Results

In total, 345 participants expressed interest and were screened for the study, of whom 269 (78%) were enrolled. Of those enrolled, most were overweight or obese (91%), African American (94.4%), and women (85.1%), with a mean age of 44.3 (SD, 12.2) years (Table 1). When considering hypertension status, 113 (42%) had a self-reported diagnosis of hypertension, of whom 95 (84%) reported taking prescribed antihypertensive medication; 97 (36%) had no self-reported medical history of hypertension, yet when blood pressure was measured had a clinical diagnosis of prehypertension or hypertension (ie, undiagnosed subgroup); and 59 (22%) had no self-reported or clinical diagnosis of hypertension. Sex, race, marital status, educational attainment, income, and HRQOL did not differ significantly by hypertension status at baseline. However, participants with self-reported diagnosis of hypertension were older, had a higher body mass index, and were more likely to self-report a diagnosis of high blood glucose or high cholesterol. Those with no self-reported or clinical diagnosis of hypertension were more likely to be current smokers.

**Table 1:** Characteristics of Hub City Steps Participants and Comparisons Between Subgroups of Hypertension Status at Baseline, Mississippi, 2010

Characteristic	All Participants (N = 269)	Self-Reported Diagnosis of Hypertension (Group A) (n = 113)	No Self-Reported Hypertension, But Clinical Diagnosis of Prehypertension or Hypertension (Group B) (n = 97)^a^	No Self-Reported or Clinical Diagnosis of Prehypertension or Hypertension (Group C) (n = 59)	*P* b
**Sex, n (%)**					
Male	40 (14.9)	19 (16.8)	17 (17.5)	4 (6.8)	.14
Female	229 (85.1)	94 (83.2)	80 (82.5)	55 (93.2)	
**Race,^c^ n (%)**					
African American	254 (94.4)	108 (95.6)	91 (93.8)	55 (93.2)	.77
White	14 (5.2)	4 (3.5)	6 (6.2)	4 (6.8)	
American Indian/Alaska Native	1 (0.4)	1 (0.9)	0 (0.0)	0 (0.0)	
**Marital status,^d^ n (%)**					
Married	113 (42.0)	44 (38.9)	49 (50.5)	20 (33.9)	.09
Widowed	12 (4.5)	10 (8.9)	1 (1.0)	1 (1.7)	
Divorced	47 (17.5)	20 (17.7)	13 (13.4)	14 (23.7)	
Separated	8 (3.0)	6 (5.3)	0 (0.0)	2 (3.4)	
Never married	89 (33.1)	33 (29.2)	34 (35.1)	22 (37.3)	
**Education,^e^ n (%)**					
Less than high school graduate	12 (4.5)	7 (6.2)	2 (2.1)	3 (5.1)	.06
High school graduate or general equivalency diploma	41 (15.2)	23 (20.4)	12 (12.4)	6 (10.2)	
Some college	74 (22.7)	24 (21.2)	30 (30.9)	20 (33.9)	
College degree or higher	142 (28.3)	59 (52.2)	53 (54.6)	30 (50.9)	
**Annual household income, $,^f^ n (%)**					
<10,000	40 (14.9)	22 (19.5)	8 (8.3)	10 (16.9)	.11
10,000–19,999	36 (13.4)	16 (14.2)	12 (12.5)	8 (13.6)	
20,000–29,999	54 (20.1)	20 (17.7)	22 (22.9)	12 (20.3)	
30,000–39,999	37 (13.8)	14 (12.4)	14 (14.6)	9 (15.3)	
40,000–49,999	30 (11.2)	13 (11.5)	10 (10.4)	7 (11.9)	
≥50,000	71 (26.5)	28 (24.8)	30 (31.3)	13 (22.0)	
**Other self-reported cardiovascular disease risk factors, n (%)**					
Current smoker	23 (8.6)	9 (8.0)	4 (4.1)	10 (17.0)	.02^g^
Diagnosed high blood glucose	42 (15.6)	30 (26.6)	6 (6.2)	6 (10.2)	<.001^h^
Diagnosed high cholesterol	52 (19.3)	33 (29.2)	14 (14.4)	5 (8.5)	.002^h^
**Age, y, mean (SD)**	44.3 (12.2)	48.6 (12.0)	41.8 (11.8)	39.9 (10.5)	<.001^h^
**Body mass index (kg/m^2^), mean (SD)**	34.7 (8.1)	36.1 (8.6)	34.2 (8.0)	32.7 (7.0)	.03^i^
**Health-related quality of life,^j^ mean (SD)**	2.9 (0.9)	3.1 (0.9)	2.9 (0.8)	2.8 (1.0)	.08

Abbreviation: SD, standard deviation.

a No self-reported medical history of hypertension at baseline, but had a measured systolic blood pressure >120 mm Hg or diastolic blood pressure >80 mm Hg (ie, clinical prehypertension or hypertension).

b *P* value for differences among groups.

c Categories collapsed to African American vs others for comparison purposes.

d Categories collapsed to married vs others for comparison purposes.

e Categories collapsed for comparison purposes to less than high school or high school graduate vs some college or college graduate.

f Treated as continuous variable (12 categories in $5,000-step increments).

g Pairwise comparison: group B < group C.

h Pairwise comparison: groups A > groups B and C.

i Pairwise comparison: group A > group C.

j Self-reported health status, treated as continuous variable (1 = excellent, 5 = poor).

Table 2 details blood pressure changes by subgroup of hypertension status at baseline and, separately, changes by excluding those who had antihypertensive medication changes. Across both the systolic and diastolic models, blood pressure significantly improved from baseline to 6 months for participants with self-reported hypertension and those with undiagnosed hypertension, but not among those with no hypertension. There was a significant group effect (*P* < .001), such that participants with self-reported hypertension and those with undiagnosed hypertension experienced significantly greater improvements in SBP compared with the subgroup with no hypertension. Subgroup analyses were similar for the DBP models, with the exception that participants with undiagnosed hypertension also experienced significantly greater improvements compared with the other 2 subgroups. The nonsignificant time effect indicates that the decreases apparent at 3 months were not different from those at 6 months. When the 8 participants who reported a change in antihypertensive medication use were removed from the data set, interpretation of the subgroup analysis did not change.

**Table 2:** Blood Pressure Changes by Subgroup of Hypertension Status at Baseline and Antihypertensive Medication Changes, Mississippi, 2010

Hypertension Group	n^a^	Baseline Value (n = 269), Mean (SD)	Change From Baseline to 3 Months (n = 227), Mean (SD)^b^^,^ c	Change From Baseline to 6 Months (n = 190), Mean (SD)^b^^,^ d	*P* time^e^	*P* group
**All participants with available data**						
**Systolic blood pressure**						
Self-reported diagnosis of hypertension (group A)	113	132.4 (18.9)	**−7.5 (16.1)**	−**8.2 (18.2)**	.45	<.001^f^
No self-reported, yet clinical diagnosis of prehypertension or hypertension (group B)^g^	97	131.1 (14.9)	**−10.8 (14.0)**	**−12.3 (16.3)**		
No diagnosis of hypertension (group C)	59	105.3 (8.5)	3.5 (12.7)	2.1 (11.1)		
**Diastolic blood pressure**						
Self-reported diagnosis of hypertension (group A)	113	86.0 (12.1)	**−2.9 (11.4)**	**−4.0 (11.9)**	.14	<.001^h^
No self-reported, yet clinical diagnosis of pre-hypertension or hypertension (group B)^g^	97	87.2 (9.7)	**−5.4 (10.6)**	**−7.8 (10.2)**		
No diagnosis of hypertension (group C)	59	71.2 (8.3)	1.6 (9.4)	0.5 (7.9)		
**Excluding 8 participants with hypertension medication changes**						
**Systolic blood pressure**						
Self-reported diagnosis of hypertension (group A)	106	131.3 (18.7)	**−7.0 (15.9)**	**−7.8 (17.6)**	.37	<.001^f^
No self-reported, yet clinical diagnosis of pre-hypertension or hypertension (group B)^g^	96	130.8 (14.7)	**−10.4 (13.7)**	**−12.3 (16.3)**		
No diagnosis of hypertension (group C)	59	105.3 (8.5)	3.5 (12.7)	2.1 (11.1)		
**Diastolic blood pressure**						
Self-reported diagnosis of hypertension (group A)	106	85.5 (11.9)	**−3.0 (10.8)**	**−3.8 (11.9)**	.36	<.001^h^
No self-reported, yet clinical diagnosis of pre-hypertension or hypertension (group B)^g^	96	87.1 (9.6)	**−5.3 (10.6)**	**−7.8 (10.2)**		
No diagnosis of hypertension (group C)	59	71.2 (8.3)	1.6 (9.4)	0.5 (7.9)		

Abbreviation: SD, standard deviation.

a Participant categorization at baseline.

b Bolded values represents significant changes from baseline at *P* ≤ .05.

c At 3 months, retention rates were 87% (98/113), 81% (79/97), and 85% (50/59) for groups A, B, and C, respectively.

d At 6 months, retention rates were 73% (83/113), 66% (64/97), and 73% (43/59) for groups A, B, and C, respectively.

e Change from baseline to 3 months compared with change from baseline to 6 months.

f Pairwise comparison: groups A and B < group C.

g No self-reported medical history of hypertension at baseline, but had a measured systolic blood pressure >120 mm Hg or diastolic blood pressure >80 mm Hg (ie, clinical prehypertension or hypertension).

h Pairwise comparison: B < A < C.

When controlling for time, age, marital status, education status, income, and baseline body mass index, differences in blood pressure changes among subgroups remained significant with magnitudes greatest for the undiagnosed hypertension subgroup (SBP, −13.9; DBP, −6.4), followed by the subgroup with self-reported diagnosis of hypertension (SBP, −7.9; DBP, −3.4). Because time was not significant in these models, the changes represent average (3 months and 6 months together) intervention changes. Although none of the covariates were significant in the DBP model, age, income, body mass index, and education were significant in the SBP model. Average decreases in SBP were 2.0, 0.6, and 0.3 mm Hg, respectively, for every 10 years increase in age, 1 unit ($5,000) increase in income, and 1 kg/m^2^ increase in body mass index at baseline. Furthermore, both education groups had significant average decreases in SBP, though the magnitude of change in the group with less than high school or high school graduate level of education (9.1 mm Hg) was significantly larger than the magnitude of change in the group with some college or a college degree (4.9 mm Hg).

Health-related quality of life improved by 0.2 (standard error of mean [SEM], 0.06; *P* < .001), 0.2 (SEM, 0.07; *P* = .02), and 0.3 (SEM, 0.08; *P* = .002) points, respectively, for the self-reported hypertension, undiagnosed hypertension, and no hypertension subgroups. In the HRQOL model, improvements apparent at 3 months were not different from those at 6 months; therefore, these reported changes represent average intervention changes. Changes among hypertension subgroups were not significantly different from one another.

## Discussion

Effective strategies are needed to recruit, engage, and intervene with people who have undetected and uncontrolled hypertension (21). These secondary analyses of participants’ baseline hypertension status from a CBPR-guided lifestyle intervention reveal findings that can inform potential solutions for addressing the silent hypertension epidemic. Although recruitment and inclusion criteria were not restricted to people with hypertension, the prevalence of hypertension in the enrolled sample was 78%, of whom 42% had self-reported hypertension and 36% were unaware of their clinical diagnosis of prehypertension or hypertension. Although it is difficult to make precise comparisons because of methodologic differences, the hypertension prevalence and lack of awareness found in Hub City Steps are about double the Forrest County estimates for hypertension prevalence (ie, 44% women, 40% men) and unawareness (ie, 18% for both women and men) (6). This finding suggests that setting unrestrictive eligibility criteria and using recruitment strategies that focused more broadly on the health and social opportunities of programs were effective approaches for reaching people at risk.

The 36% who were unaware of their hypertension experienced the largest blood pressure improvements, indicating those most in need of effective hypertension treatment benefited the most from this lifestyle intervention. Additionally, most (84%) of those with self-reported hypertension reported prescribed antihypertensive medication use, yet also saw significant improvements over the course of the intervention. Because the statistical or clinical interpretations did not change after excluding the low proportion of participants that reported changes in antihypertensive medication, it is reasonable to assume that medication change did not confound the significant blood pressure reductions from this lifestyle trial. Given the persistence of hypertension unawareness and problems with medical care access in health-disparate regions, it is unlikely that primary care–based treatment approaches alone can adequately reach and engage populations in most need of behavioral lifestyle changes to mitigate hypertension risk factors (22).

All subgroups had improvement in HRQOL, even the subgroup without hypertension that did not otherwise benefit from blood pressure improvements. This finding is congruent with other studies that have demonstrated improvements in quality of life among participants enrolled in behavioral lifestyle interventions (23,24). Quality of life is an effectiveness indicator that can provide a critical participant-centered evaluation indicating the effect of program delivery practices (13).

If the CBPR efforts of the Hub City Steps lifestyle intervention had applied a recruitment and inclusion or exclusion protocol similar to PREMIER and DASH, the success at reaching those with undetected hypertension or those with treated but uncontrolled hypertension may have been diminished. Both the PREMIER and DASH trials provide effective strategies for the treatment and control of hypertension (25–27). These nonpharmacological, lifestyle trials were originally tested through clinical or primary care settings and under highly controlled conditions with strict participant inclusion and exclusion criteria (eg, antihypertensive medication use was an exclusion criterion). A number of CBPR trials have begun to translate evidence-based hypertension interventions into community-based settings, with success in enrolling both women and men (28,29). However, there remains a great need to understand effective strategies and protocols to promote recruitment and engagement of community residents into programs that can assist with hypertension control, especially for people who are either unaware of their hypertension status or who are aware but lack control (4,21).

This study is not without limitations: it was not specifically designed or powered to detect effects based on subgroup analysis. For example, race, sex, and smoking status could not be used as covariates in the multivariable models because of small sample sizes. However, the *P* values presented in Table 2 indicated sufficient power to detect effects among post-hoc groups and lessen concerns that may have existed if the analysis had revealed null effects. Nonetheless, the high proportion of women limits generalizability of findings. Furthermore, the classifications for hypertension status were made based on a single day’s measure and not based on at least 2 high readings at 2 different times, the suggested standard for diagnosis (30). Finally, only 1 of 4 items from the CDC’s Healthy Days measure was used to measure HRQOL (19). Although this may limit generalizability and comparison with other studies, it does not diminish the significant preintervention–postintervention improvements realized for this intervention. Additional analytical efforts are under way to understand changes in other program outcomes, such as diet and physical activity, and to examine how program adherence influences changes in outcomes.

Evaluation of culturally appropriate community-based efforts is needed to understand how to reach the high proportion of people with undetected and uncontrolled hypertension. The Hub City Steps initiative provides insight into use of such approaches to reach and enroll people in effective lifestyle programs for hypertension control. Recognizing the significant improvements realized in blood pressure and HRQOL in this intervention, others may be able to apply our CBPR approach to guide community-driven efforts addressing hypertension.
